# Soil acidification and salinity: the importance of biochar application to agricultural soils

**DOI:** 10.3389/fpls.2023.1206820

**Published:** 2023-09-14

**Authors:** Kai Huang, Mingquan Li, Rongpeng Li, Fahd Rasul, Sobia Shahzad, Changhong Wu, Jinhua Shao, Guoqin Huang, Ronghui Li, Saad Almari, Mohamed Hashem, Muhammad Aamer

**Affiliations:** ^1^ China Guangxi Key Laboratory of Water Engineering Materials and Structures, Guangxi Hydraulic Research Institute, Nanning, China; ^2^ Department of Agronomy, University of Agriculture Faisalabad, Faisalabad, Pakistan; ^3^ Islamia University of Bahawalpur, Bahawalnagar, Pakistan; ^4^ Research Center on Ecological Sciences, Jiangxi Agricultural University, Nanchang, China; ^5^ College of Civil Engineering and Architecture, Guangxi University, Nanning, China; ^6^ King Khalid University, College of Science, Department of Biology, Abha, Saudi Arabia

**Keywords:** biochar, acidic soils, microbial activity, nutrient uptake, salinity, toxic ions

## Abstract

Soil acidity is a serious problem in agricultural lands as it directly affects the soil, crop production, and human health. Soil acidification in agricultural lands occurs due to the release of protons (H^+^) from the transforming reactions of various carbon, nitrogen, and sulfur-containing compounds. The use of biochar (BC) has emerged as an excellent tool to manage soil acidity owing to its alkaline nature and its appreciable ability to improve the soil’s physical, chemical, and biological properties. The application of BC to acidic soils improves soil pH, soil organic matter (SOM), cation exchange capacity (CEC), nutrient uptake, microbial activity and diversity, and enzyme activities which mitigate the adverse impacts of acidity on plants. Further, BC application also reduce the concentration of H^+^ and Al^3+^ ions and other toxic metals which mitigate the soil acidity and supports plant growth. Similarly, soil salinity (SS) is also a serious concern across the globe and it has a direct impact on global production and food security. Due to its appreciable liming potential BC is also an important amendment to mitigate the adverse impacts of SS. The addition of BC to saline soils improves nutrient homeostasis, nutrient uptake, SOM, CEC, soil microbial activity, enzymatic activity, and water uptake and reduces the accumulation of toxic ions sodium (Na^+^ and chloride (Cl^-^). All these BC-mediated changes support plant growth by improving antioxidant activity, photosynthesis efficiency, stomata working, and decrease oxidative damage in plants. Thus, in the present review, we discussed the various mechanisms through which BC improves the soil properties and microbial and enzymatic activities to counter acidity and salinity problems. The present review will increase the existing knowledge about the role of BC to mitigate soil acidity and salinity problems. This will also provide new suggestions to readers on how this knowledge can be used to ameliorate acidic and saline soils.

## Introduction

Soil acidification is a natural process that occurs at a very slow rate during soil weathering, however, anthropogenic activities like intensive agricultural practices can speed up this process (Bolan et al., 2023). In agricultural soils, the continuous application of nitrogen (N) and sulfur (S) increases the concentration of H^+^ ions which negatively affect plant growth, soil properties, and microbial activities (Fageria and Nascente, 2014). The increased H^+^ concentration increases the mobility and solubility of toxic metals aluminum (Al) and manganese (Mn) that natively affect plant growth and development and human health by entering the food chain (Briffa et al., 2020). Besides this soil acidity also reduces the availability of essential nutrients [(phosphorus (P), molybdenum (Mo), calcium (Ca), and magnesium (Mg)] thus negatively affecting the plant’s growth and development (Ritchie, 1989; Briffa et al., 2020; Cui et al., 2020). Soil acidification is a very slow process that occurs through weathering of minerals and rainfall that increased the loss of basic cations. Yet, the application of high rates of nitrogen (N) fertilizers, acid rains, and industrial climate conditions are the main reasons of a substantial increase in soil acidification around the globe (Guo et al., 2010).

Soil salinity is a dangerous stress that hinders plant growth by altering plant morphological, physio-biochemical, and molecular processes (Zörb et al., 2019; Hoque et al., 2022a; Imran et al., 2022; Khan et al., 2023). Every year around the globe 1-2% of cultivated soils are reduced due to salinity and about 23% of arable land (800 million hectares) is salt affected which is a serious threat to food production (Alqahtani et al., 2019). Besides this, it has been estimated that 50% of arable lands will be converted into salt-affected soil by the end of 2050 owing to an increase in groundwater levels with high concentrations of salts, inefficient irrigation, and drainage systems, and overuse of chemical fertilizers (Shahid et al., 2018; Raza et al., 2022). Salinity stress causes a substantial reduction in yield and it has been documented that this stress can cause yield losses of up-to 65% in many cultivated areas (Farahmand and Sadeghi, 2020; Chattha et al., 2022; Nawaz et al., 2022). Plants grown under saline conditions face a reduction in germination, seedling growth, and yield and change in physiological and molecular processes (Khan et al., 2021; Hassan et al., 2022). Salinity also induced the production of reactive species (ROS) that damage protein, deoxyribonucleic acid (DNA), and lipids and increase the loss of important osmolytes (Jiang et al., 2020; Kamran et al., 2020). Besides this salinity stress also affect plant growth and development by inducing ionic and osmotic stress (Liang et al., 2022). The salinity-induced ionic toxicity increases the concentration of toxic ions and decreases the concentration of essential nutrients like calcium (Ca) and potassium (K) (Akhtar et al., 2015a). On the other hand under salinity-induced osmotic stress soil water is rapidly decreased due to the considerable reduction in water potential of soils owing to an increase in salt concentration (Zhang et al., 2023) which consequently reduced plant growth by decreasing nutrient and water uptake and photosynthetic rate (Hussain et al., 2019).

Globally, different liming materials including lime, dolomite, and steel slag are being used to manage the soil acidity problem. Recently biochar (BC) has also emerged as an excellent liming material to manage soil acidity (Shi et al., 2019), soil salinity and heavy metals polluted soils (Mehmood et al., 2021; Mehmood et al., 2022a; Mehmood et al., 2022b; Mehmood et al., 2023). BC addition to acid soils increases soil pH which improved plant growth and plant responses (Rondon et al., 2007; Shi et al., 2019; Deus et al., 2020). BC application also changes the soil properties (pH, porosity, and redox state) and improves the mobilization of nutrients that favors plant growth under acid soils (Major et al., 2010; Borchard et al., 2012). BC could be an important alternative to lime to amend acidic soils and results of previous studies showed that an increase in crop productivity by BC under acidic soils is caused by the liming effect of BC (Jeffery et al., 2011). Besides this increase in soil pH also decreases the bio-availability of Al and fixation of P by iron (Fe) and Al cations (Fe^3+^ and Al^3+^) (Cui et al., 2011). Biochar application also reduce the ROS production, MDA accumulation (Pandit et al., 2018) and it increased the uptake of nutrients, photosynthesis, plant water relations which improve the plant growth in acidic soils (Yan et al., 2021). Thus, BC application could be an important practice to improve the availability of nutrients and decrease the availability of toxic metals to improve plant growth under P in acidic soils (Zhang et al., 2022a).

To reduce the toxic effects of salinity scientists are using different techniques such as gypsum, humic substances, sulfur, organic amendments, green manures, and salt-tolerant crops (Meena et al., 2020; Shilev, 2020). The use of organic amendments has emerged as an excellent tool to cope with salinity stress. Among organic amendments, recently BC got considerable attention around the globe to solve the problem of salinity stress (Shilev, 2020). BC reduces the toxic effects of salinity by increasing antioxidant activities, photosynthetic efficiency, plant water relations, accumulation of osmolytes, hormones, and secondary metabolites, and decreasing ROS production in plants (Parkash and Singh, 2020; Kerbab et al., 2021).

Therefore, in the present review, we discussed the liming potential of BC to address the acidity and salinity problems. We have focused on the effect of soil acidity and salinity on plants followed by the role of biochar to mitigate the adverse effect of acidity and salinity. We have discussed various mechanisms by which BC reduces the toxic effects of acidity and salinity. Particularly, we have focused on how BC affects soil properties to manage acidity and salinity problems. We believe that the present review would fulfill the knowledge gaps on the liming capacity of BC. The increased knowledge about the liming capacity of BC will benefit BC and other agriculture industries to search out the potential of BC and other carbon compounds to manage the acidity and salinity problems.

### Effects of soil acidity plants and soil

Soil acidification is a serious threat to sustaining crop production and acid soils cover 30-40% of arable lands globally (Misra and Tyler, 1999; Kochian et al., 2004). Soil pH has a significant impact on plants owing to the fact it affects every aspect of nutrients taken by plants. However, in acidic soils, plants face three major toxicity Al^3+^, Mn^2+^, and H^+^ which inhibit root growth, cell division, and nutrient uptake and cause modification of the cytoskeleton (Bojórquez-Quintal et al., 2017; Kaur et al., 2019). In many cases there is no obvious effect of Al toxicity, instead, these effects are manifest as P deficiency symptoms with dark green leaves, stunted growth, late maturity, and purpling of stems, leaves, and veins (Kaur et al., 2019). Mn is the second toxic metal in acid soils, although Mn is an essential nutrient for plants, however, it becomes toxic when plants take it in excess (Sumner et al., 1991). The low soil pH is often linked with inhibited root growth owing to H^+^ influx in roots (Yang et al., 2005). The higher H^+^ influx causes membrane depolarization and also affects the acidity of the cytoplasm (Babourina et al., 2001). Besides this high H^+^ also adversely affects the root tissues which causes a substantial reduction in growth and development (Msimbira and Smith, 2020).

Acidic soils also affect the uptake of phosphorus, root length, and diameter of roots (Robles-Aguilar et al., 2019). Further, low soil pH induced ROS production (Song et al., 2011) which oxidizes the cellular ultrastructure and causes oxidative damage to cellular organelles (Sharma et al., 2012). For example, Zhang et al. (2015) also noted a substantial increase in lipid peroxidation and concentration of hydrogen peroxide (H_2_O_2_) with an increase increasing H^+^ co\ncentration in the growth medium, while Martins et al. (2013) also found an increase in lipid peroxidation of *Plantago* plants growing in pH 4 soil. Yang et al. (2011) found that low soil pH increased the membrane permeability of Eucalyptus plant leaves. In another study, Tóth et al. (2020) reported a significant increase in MDA, proline, and antioxidant activities at a soil pH of 5. Further, these authors also reported that soil pH and the growth stage of the plant also affect the MDA accumulation and antioxidant activities of plants (Tóth et al., 2020). Another study conducted on soybean showed that soil pH below 5.2 does not favor plant growth and results in a substantial reduction in plant growth (Bakari et al., 2020). Soil pH <5 also limits the nodulation owing to Al and Fe toxicity that induces poor formation and functioning of nodules (Nisa et al., 2012).

Soil acidification also increases the concentration of toxic metals (Fe and Al) which enhanced the retention of P in the soil through adsorption and precipitation which in turn reduces plant growth (Ng et al., 2022). Soil acidity also increases the deficiency of base cations (Ca^2+^ and Mg^2+^) by causing the leaching of exchangeable Ca^2+^ and Mg^2+^ (Maathuis, 2009). Nonetheless, low soil pH may not affect the zinc (Zn) for plant growth, however, a decrease in soil pH also increases Mn concentration which adversely affects crops and many crops are sensitive to high Mn concentration (Senbayram et al., 2015; Alejandro et al., 2020). Generally, Fe has low solubility in acidified soils (Zhu et al., 2021) therefore redox plays a crucial role in solubilizing Fe to meet the plant needs (Jin et al., 2014). Moreover, it has been reported that soil acidity also increases availability which also affects plant growth (Desa and Ernani, 2016).

Soil acidity adversely impacts synergistic interaction amid the legume crops and their linked rhizobia. A soil pH lower than 6 reduces the nodulation which in turn reduces N fixation. The available Ca and Mo are considered to be essential for N fixation, however, in acidic soils both these nutrients become deficient which reduces the N fixation and subsequent growth in plants (Ferguson et al., 2013). On the other hand soil acidity also increase Al and Mn which induced the malformation and malfunctioning of root systems thus decreasing the nutrient and water uptake and negatively affecting plant growth and development (Pavlů et al., 2021). Moreover, soil acidification also affects the residence time and leaching potential of trace metals and a decrease in soil pH can increase the amount the trace elements in leachates (Taylor, 1975). The decrease in soil pH also affects the release of cadmium (Cd), for instance, decreased soil pH to 2.8 results in 85% release of Cd through leaching while decreased pH often increases the adsorption of As-V, and decrease adsorption of As-III which consequently affects plant growth (Rahman et al., 2019).

### Biochar an important player to manage soil acidity

**Figure 1:**
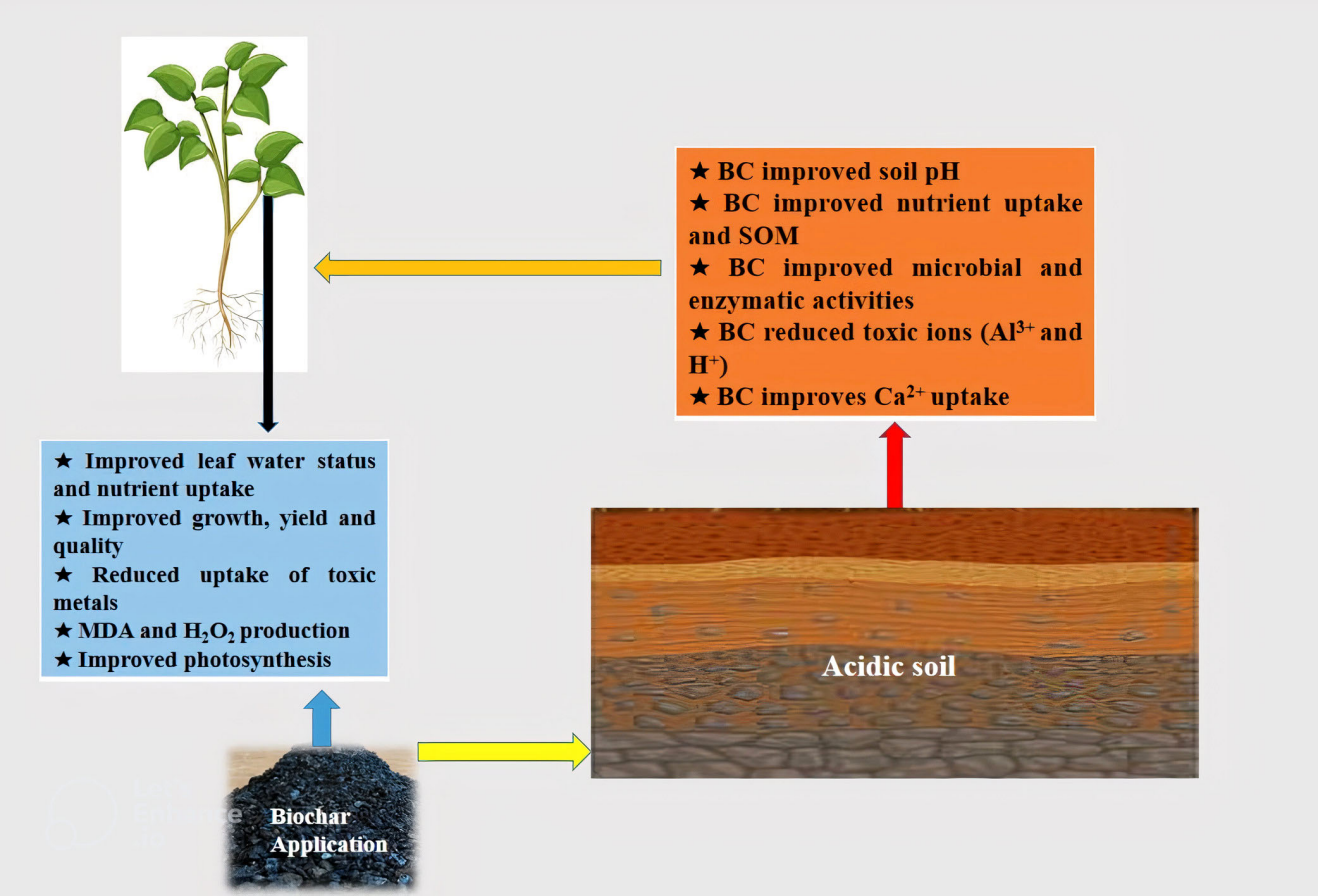
The application of BC to acid soils improves soil pH, nutrient uptake, microbial and enzymatic activities and reduce the uptake of toxic metals that improved the growth and yield by reducing MDA and H_2_O_2_ production and increasing antioxidant activities and photosynthetic efficiency.

Lime is an important material used globally to manage acidic soils; however, high cost and limited availability limit its use in many areas (Tully et al., 2015; Frimpong-Manso et al., 2020). In this context, BC produced from agricultural waste like rice husk and corn cob can provide suitable liming material to tackle soil acidity (Rondon et al., 2007). BC has appreciable potential to sequester soil carbon owing to its stable nature and it also improves soil physicochemical properties (**Figure 1**) which improved soil fertility and productivity (Bolan et al., 2021). BC-induced induce direct and indirect impacts on acidic soils and the former can be get by improved physicochemical and biological properties while the latter can be achieved by mobilization of essential nutrients and immobilization of toxic metals.

### Biochar improves physico-chemical properties soils to counter soil acidity

Soil pH, cation exchange sites, and electrolyte concentrations are important factors that affect the surface charge of soil particles. Likewise, soil pH also affects the dispersion and sensitivity of soil hydraulic parameters (**Table 1**), therefore, the composition and quantity of organic matter (OM) play an important role in determining the extent of pH influence (Ali et al., 2019; Wen et al., 2020). The liming materials change the soil pH and Ca concentration which affect the flocculation/dispersion in soils. Though, the dispersion of clay minerals can be significantly decreased by an increase in Ca percentage due to a decrease in density charge (Junior et al., 2020). Additionally, liming substances provide an adequate amount of Ca which plays an important role in soil aggregate stability (Junior et al., 2020). The OH- ions produced from the addition of BC can neutralize the H+ ions, thus they can reduce the mobility as well as bioavailability of Al^3+^ and Mn^2+^. Moreover, the addition of BC to acidic soils also increases the solubility of P and Mo and addresses the deficiency of Ca^2+^ and Mg^2+^ which in turn increase the plant biomass under acidic soils (Alburquerque et al., 2014; Zhang et al., 2022b).

**Table 1:** Effect of biochar application on soil physiochemical and biological properties of acidic soils.

Biochar application	Field/lab study	Soil acidity (pH)	Effects	References
RSBC 40 t ha^-1^	Field	5.18	BC application increased soil pH, dissolved organic carbon, microbial biomass carbon, nitrogen and abundance of nosZ and nirk genes	Aamer et al., 2021
RSBC, MSBC, WSBC, RHBC, BBC 22.5 t ha^-1^	Field	4.78	Biochar increased soil aggregates and water retention capacity.	
Maize stalk biochar	Pot	5.76	BC application enhanced soil pH, total N, Total P, ${\text{NO}}_{3}{-}^{\;}$ ^-^-N, C/N ratio, BC also increased the soil fungal ITS genes copy numbers.	Yao et al., 2017
Senna *siamea biochar* (BC)	Field	5.40	BC with less dose of NPK: improved the soil pH, CEC, available phosphorus, while BC with high rate of NPK enhanced bacterial and fungal population, microbial biomass carbon and basal respiration rates.	Phares et al., 2022
Modified wheat straw biochar	Lab	5.69	Higher dose of BC application enlarged soil pH, EC, SOC, ${\text{NH}}_{4}{\;}^{+}-\text{N}$ and ${\text{NO}}_{3}{\;}^{-}$ -N.	Khan et al., 2022
RSBC 1500kg/ha	Field	6.05	Low dose of Biochar application with reduced NPK fertilizer increased soil pH, available NP, SOC, and it reduced soil bulk density	An et al., 2022
Pine chip biochar and Poultry litter biochar	Lab study	5.64	BC amended enhanced soil pH, total SOC, and ${\text{NO}}_{3}{\;}^{-}$ -N. while poultry litter biochar decreased MCB.	
RSBC 67.2 t ha^-1^	Lab study	5.21	BC application in red soil increased the soil pH, microbial biomass carbon, ${\text{NO}}_{3}{-}^{\;}$ -N, genes abundance (nosZ, nirK, AOA, and AOB), and urease (UR) enzymatic activities and reduced ${\text{NH}}_{4}{+}^{\;}-\text{N}$ and the activity of nitrate reductases.	
Peanut shell biochar (PBC)	Pot	4.41	The application of PBC improved the pH, CEC, water-soluble ${\text{SO}}_{4}{\;}^{2-}$ , and dissolved organic carbon DOC in the paddy soil	Chao et al., 2018
Pinus bark biochar	Lab study	4.76	BC application improved the soil pH, exchangeable cations, and decreased soil exchangeable acidity and exchangeable aluminum	Zhao et al., 2015

RSBC, rice straw biochar; MSBC, maize straw biochar; WSBC, wheat straw biochar; RHBC, rice husk biochar; BBC, bamboo biochar; CEC, cation exchange capacity.

In another study, it was noted that the application of BC prepared from peanut shells and cattle manure substantially reduced the Al toxicity by increasing soil pH and availability of nutrients (Lin et al., 2018). Moreover, these authors also noted that an increase in soil pH following BC application resulted in a significant decrease in exchangeable Al and H ions and an increase in exchangeable K, Na, Ca, Mg, and cation exchange capacity (CEC). Nonetheless, the increase in soil pH was much great in cattle manure BC as compared to peanut shell BC (Lin et al., 2018). The increase in soil pH following BC application also varied with pyrolysis temperature and the effect of BC prepare at high temperatures is greater than those of BC prepared at low temperatures (Sani et al., 2020). As the pyrolysis temperature increases, the degree of dehydration and decomposition of organic acids in OM increases which increases the concentration of basic groups (Sani et al., 2020). In another study, cow dung BC improved the acidic soils as compared to BC made from peanut shells (Geng et al., 2022). Further, the increase in pyrolysis temperature also increased the concentration of K, Na, Ca, Mg, and other mineral elements (Das et al., 2021) and it also increases the acidification ability of BC. BC application further increased the concentration of P, K, and Mg concentrations which in turn improved the above-ground biomass in acidic soils. The improved soil pH following BC application improved the availability of P, K, and Mg and decreased the Mn and copper (Cu) concentrations which improve the overall above-ground biomass and quality of plants under acidic soils (Yan et al., 2021). To summarize, BC improves nutrient uptake, SOM, CEC and reduce the uptake of toxic ions that helps to counter the effect of soil acidity.

### Biochar improves biological properties of soil to counter acidity

Biochar has direct and indirect impacts on soil microbes, and it has been reported that BC application improved the availability of K by increasing the activity and number of *Azotobacter* and *Pseudomonas* in acidic soils (Zhang et al., 2022b). Soil pH, OM, and ECE are the most important factors that affect the fungal and bacterial community and BC application has been reported to increase the community of both fungal and bacterial communities (**Table 2**). It has been reported that BC application substantially increased the abundance of *Pseudarthrobacter*, AMF, and endophytic bacteria which improved the growth (Zhang et al., 2022b). In addition, BC application also increased the mineralization of N, P, and S while BC also enhanced the fixation of N in acidic soils. The application of liming increases the mineralization of nutrients by increasing their occurrence in soil solution for the uptake of plants (Bossolani et al., 2020). Besides this, BC also provides base cation for rhizobia legumes which increases the nodulation as well as N fixation in acidic soils (Zhang et al., 2021).

**Table 2:** Effect of biochar application on growth, yield, physiological and biochemical responses of different crops under acidic soils.

Biochar application	Crop/Field/lab study	Soil acidity (pH)	Effects	References
Eucalyptus BC 20 t ha^-1^	Rice (Pot)	5.96	BC application improved plant height, root and shoot growth and biomass production	Shetty and Prakash, 2020
RSBC, MSBC, WSBC, RHBC, BBC 22.5 t ha^-1^	Rice,Brassica napus and maize (Field)	4.78	Five different types of biochar increased rice, rape and maize yield in consecutive cropping season.	
*Senna siamea* biochar (BC)	Maize (Field)	5.40	BC combined with less dose of NPK: improved the maize yield in both years of experiment.	Phares et al., 2022
RSBC 1500kg/ha	Rice (Field experiment)	6.05	Biochar application with reduced NPK fertilizer increased grain yield, NP in grains and straw, root biomass	An et al., 2022
RSBC 40 t ha^-1^	Rice (Field experiment)	5.18	BC application with nitrogenous fertilizer increased no. of tillers, plant height, paddy yield and biomass yield	Aamer et al., 2021
Bamboo biochar 5%	Tea (pot study)	4.33	BC addition improved plant P, K and Mg concentrations, above ground biomass and photosynthesis rate.	Yan et al., 2021
*Artemisia vulgaris* derived biochar 10 t/ha	Maize and black gram (Lab study)	5.24	BC increased the seedling germination, root/shoot length, coleoptile length, weight and shoot biomass in maize and black gram	Das et al., 2020
Red gram stalk biochar 5 t/ha	Black gram (field study)	5.7	BC application with phosphobacteria increased root length, root nodulation, plant height, stomatal conductance, leaf area, seed production and dry biomass production.	Kannan et al., 2021
Rice straw biochar 22.5 Mg ha^-1^	Wheat and millet (pot study)	4.84	BC incorporation increased the grain and straw yield, above ground biomass and nutrients uptake.	He et al., 2023
*Eupatorium adenophorum* weed biochar 2% (w/w)	Maize (pot study)	4.5	BC enhanced plant available phosphorus, stomatal conductance and above ground biomass.	Pandit et al., 2018

The liming ability of BC also results in the successful colonization of earthworms in crops, and the higher density of earthworm also affect the structure and aggregate stability of soils (Hirth et al., 2009). Moreover, liming also improved soil enzymatic activities and microbial biomass, and increased production of polysaccharides from improved microbial activity improved the soil aggregate stability (Fuentes et al., 2006). In another study, it was noted that BC application to acidic soils substantially improved the bacterial community structure and subsequent plant growth (Zhang J. et al., 2019). Further, these authors reported that combined BC and fertilizer application enhanced the relative abundance of some beneficial bacteria in *Oxalobacteraceae*. Further, BC also improved the abundance of *Chitinophagaceae*, *Comamonadaceae*, and *Geobacteraceae* which improved the nutrient cycling and degradation of plant residues and metal tolerance (Zhang J. et al., 2019). Likewise, another group of authors also found that BC application also increased the abundance of *Blastocatellaceae* and Acidobacteria to counter the effects of soil acidity (Pascual et al., 2015; Wang et al., 2016). Moreover, Geng et al. (2022) found that BC application to black soil enhanced the relative abundance of *Acidobacteria* and *Olpidiomycota* in acidic soils. Further, these authors also noted the significant difference in the bacterial and fungal community between the BC and without BC treatments (Geng et al., 2022). In conclusion BC mediated increase in microbial activities improves nutrient mineralization which induced positive effects on plants.

### Biochar improves soil enzymatic activities to counter soil acidity

Biochar possesses an appreciable potential to improve the soil enzymatic activity under acidic soils. The application of BC has been reported to increase the activity of urease, alkaline phosphatase, catalase, and phosphatase) with a maximum increase (45-502%) seen in the activity of catalase (CAT: Yao et al., 2021). However, Das et al. (2021) found that increasing BC application decreased the activity of acid phosphatase and these differences could be due to differences in soil characteristics, crop species, and soil properties (Geng et al., 2022). Some other authors also reported that BC application showed better results in increasing the enzymatic activity owing to the conversion of acid soils into alkaline soils following BC application (Zhang J. et al., 2019). Other authors also found that BC application not only increases the soil pH but also favors the microbial abundance and activity of soil microbes (Yuan and Xu, 2011). Further, it has been reported that BC application (30 Mg ha^-1^) also improved microbial quality and activity of α-glucosidase, acid phosphatase, arylsulfatase, and urease however, BC application greater than 30 Mg ha-1 reduced the activity of aforementioned enzymes (Lopes et al., 2021).

In another study, BC application (6 t ha^-1^) to rice field increased the carboxylate secretions, and carboxylate exudates were increased in the order of citrate > malate > acetate > oxalate (Oladele, 2019). Generally, BC application improved the enzymatic activities and it was reported that BC application improved sucrase, phosphatase, catalase, and urease activity by 63.3%, 23.2%, 50.3%, and 27.9% as compared to control and application of swine manure BC (Oladele, 2019). Likewise, Jiang et al. (2021) noticed an increase in soil carbon and nitrogen concentration by 35.4% and 34.3% respectively following the application of swine manure-based BC. In another study, it was found that BC application increased the activity of dehydrogenase, urease, and nitrate-reductase activities except for the acid phosphatase and peroxidase in cambisol and andosol. Further BC application also increased cellulose activity by 40-45% which in turn improved root growth and biomass under acidic soil (Garbuz et al., 2022). Thus, BC improves soil enzymatic activities which improve the root growth and plant functioning to counter acidity effects.

### Biochar mitigates toxic elements under acidic soils to counter acidity problem

Globally different liming amendments are used to reduce the concentration of toxic metals from acidic soils. It has been reported that BC effectively immobilizes the toxic elements including Cd, mercury (Hg), and lead (Pb) therefore, reduce their bioavailability in soils (Palansooriya et al., 2020; Xia et al., 2020). The efforts are being used globally to test the potential organic compounds to remediate the contaminated soils (Bolan et al., 2023). Since the availability of toxic metals is high in acidic soil as compared to alkaline soils, therefore, neutralizing agents are added to the soils to counter these toxic metals. The primary incentive for liming materials in acidic soils is to suppress the Al and Mn availability and BC and liming materials application is increasing to mobilize the potentially toxic metals from acidic soils. Though the effects of BC in the immobilization of toxic metals depend on BC type, soil properties, and species of potentially toxic metals (Shaheen and Tsadilas, 2010; Igalavithana et al., 2017; Igalavithana et al., 2017). Thus, BC must be carefully selected to remediate the metals contaminated soils.

Lin et al. (2018) found that BC application increased the soil pH by 0.42 units and reduced the exchangeable acid and H concentration by 52.74% and 2.86% to the control. Further, BC also reduced the active as well as exchangeable Al by 26.74% and 66.09%. These authors concluded that fresh BC could reduce Al toxicity by increasing soil pH and nutrient availability, however, aged BC had a negative effect on the reduction of Al toxicity thus inhibiting plant growth (Lin et al., 2018). The high charging density, large surface area, porosity, and presence of both polar and non-polar surface sites on BC play an important role adsorption of toxic metals along with its liming impact (Laird et al., 2010). The application of BC to can help to reduce the toxic Al toxicity through an increase in exchangeable base cation and a decrease in soil acidity (Gaskin et al., 2010; Qian et al., 2013). In another study Shetty and co-authors reported that BC application (20 t ha^-1^) reduced the soluble and exchangeable Al, therefore, reduced the toxic effects of Al on rice plants (Shetty and Prakash, 2020). In conclusion BC reduced the concentration of toxic ions by improving SOM and CEC which ensures better plant growth.

### Biochar supports the plant growth in acidic soils

Soil acidity negatively affects plant growth due to increasing in toxic metals and a reduction in the availability of nutrients. However, BC has emerged as an excellent tool to improve plant growth under acidic soils through reduced availability of toxic metals and an increase in the availability of favorable nutrients. For instance, it has been reported that BC application to acidic soils improved plant height, biomass production, and root growth by increasing soil pH and decreasing Al concentration (Lin et al., 2018) and improved soil bulk density, water holding capacity and fertilization potential (Glaser et al., 2015). Under acidic conditions excessive ROS are produced which negatively affect plant growth and development, however, plants have developed excellent antioxidant defense system to detoxify the ROS (Han et al., 2019). The application of BC substantially reduced malondialdehyde (MDA) contents (2.94-25.21%) by increasing the activity of superoxide dismutase (SOD: 1.24-23.57%) and POD (3.42-48.06%) in acidic conditions (Pandit et al., 2018). Besides this BC application under acidic conditions also increased the concentration of favorable nutrients (P, K, and M) which effectively improved the photosynthesis, leaf area, and above-ground biomass production. Further, BC application also decreases the concentration of Mn and Cu and other toxic metals which induces a positive effect on plant growth (Yan et al., 2021).

BC application also increases the available P and nitrogen use efficiency (NUE) and it also decreased the concentration of exchangeable Al which positively affect plant growth and development under acidic soils (Qiao-Hong et al., 2014). In another study, it was found that BC application to acidic soil improved the root and shoot biomass by 44.5% and 89.6% and nitrogen utilization rate by 11.08% and it also positively influence the NUE and reduced the Al concentration which led to a substantial increase in plant growth and NUE (Xia et al., 2020). In another study, it was found that BC application with phospho-bacteria significantly enhanced plant physiological parameters including leaf area, stomata conductance, and chlorophyll contents by reducing the leaf temperature. Further, BC application (5 t ha^-1^) with phosphobacteria 2 kg ha^-1^ noticed maximum organic carbon, soil available P, and P uptake by 27, 28, and 45% and the same treatment also recorded the highest yield (262 kg ha^-1^) which indicate that application of BC with phospho-bacteria is an effective practice to enhance growth and production under acidic soils (Kannan et al., 2021). Similarly, in another study conducted on acidic soil, it was found that BC application significantly improved the total soluble solids and induced positive effects on fruit quality parameters by improved soil microbial activities, soil pH, nutrient uptake and activities of urease, invertase, and catalase (Wu et al., 2020). To summarize, BC mediated improvement in plant growth in acidic soils is linked with improved SOM, nutrient uptake, CEC, microbial and enzymatic activities.

### Soil salinity effects on plants and soils

The low rainfall, high surface evaporation, increased climate change, and global temperature, movements of saline groundwater and deposition of salts from oceans are prominent reasons for soil salinity across the globe (Shrivastava and Kumar, 2015; Tavakoli and Bailey, 2017). However, the extent of these causes is increased in recent times owing to rapid industrial and economic development. Similarly intensive agricultural practices including the use of improper irrigation, fertilizers, and pesticide application are also leading to an increase in soil salinity across the globe (Bello, 2021). Of all these anthropogenic activities the excessive use of salts with poor drainage systems is the foremost factor that increases the water table and results in the deposition of salts on the soil surface (Tavakoli and Bailey, 2017).

Salinity stress can significantly reduce the growth and yield of crops by inducing, ionic, oxidative, and osmotic stresses (Taha et al., 2020). The higher concentration of Na in the growth medium causes K^+^ deficiency by increasing the exclusion of K from cells (Ma et al., 2016). Salinity also damages cellular homeostasis and denatures the proteins, lipids, and DNA and increased ROS production (Seleiman et al., 2020). Salinity-induced ROS negatively affect photosynthetic, carbon dioxide (CO_2_) uptake, relative water contents (RWC), pollen sterility, seedling and reproductive stages, therefore, negatively affect the while crop yield and quality (Alkharabsheh et al., 2021). Salinity negatively affects plant growth, however, plants’ responses to salinity stress can vary according to plant species, stage of growth, and extent of salinity stress (Al-Shareef and Tester, 2019; Alnusairi et al., 2021).

Soil salinity is a global problem and in recent times the extent of salt-affected soils is continuously increasing owing to anthropogenic activities. Aside from imposing negative effects on plant growth and also pose serious threats to soil health. For instance, salinity stress negatively affects nutrient availability, organic matter stability, and soil redox potential (Rengasamy, 2010). It has been reported that soil salinity reduces the SOM, water holding capacity, and water infiltration and disrupts the soil aggregate stability (Nan et al., 2016; Gonçalo-Filho et al., 2019).

The high concentration of Na in soil solution increases the loss of inherent soil fertility (Yu et al., 2010; Almeida et al., 2017) and it also creates osmotic potential which eventually causes cell death owing to reduced water uptake (Ahanger et al., 2018). Besides this excessive Na also causes plant wilting (Assaha et al., 2017) and it also negatively affects the soil microbial activities, microbial population, soil enzymatic activities, and biomass production (Zhang et al., 2019b). Further, soil salinity also reduced the fixation of carbon, nutrient cycling, and porosity and reduce plant growth and vigor (Cheeseman, 2015; Almeida et al., 2017). Moreover, excessive uptake of toxic ions also negatively affects plant growth by reducing the uptake and availability of water and essential nutrients including, N, P, K, Ca, Mg, Fe, and Zn (Khan et al., 2019; Safdar et al., 2019).

### Biochar an important player to alleviate soil salinity

**Figure 2:**
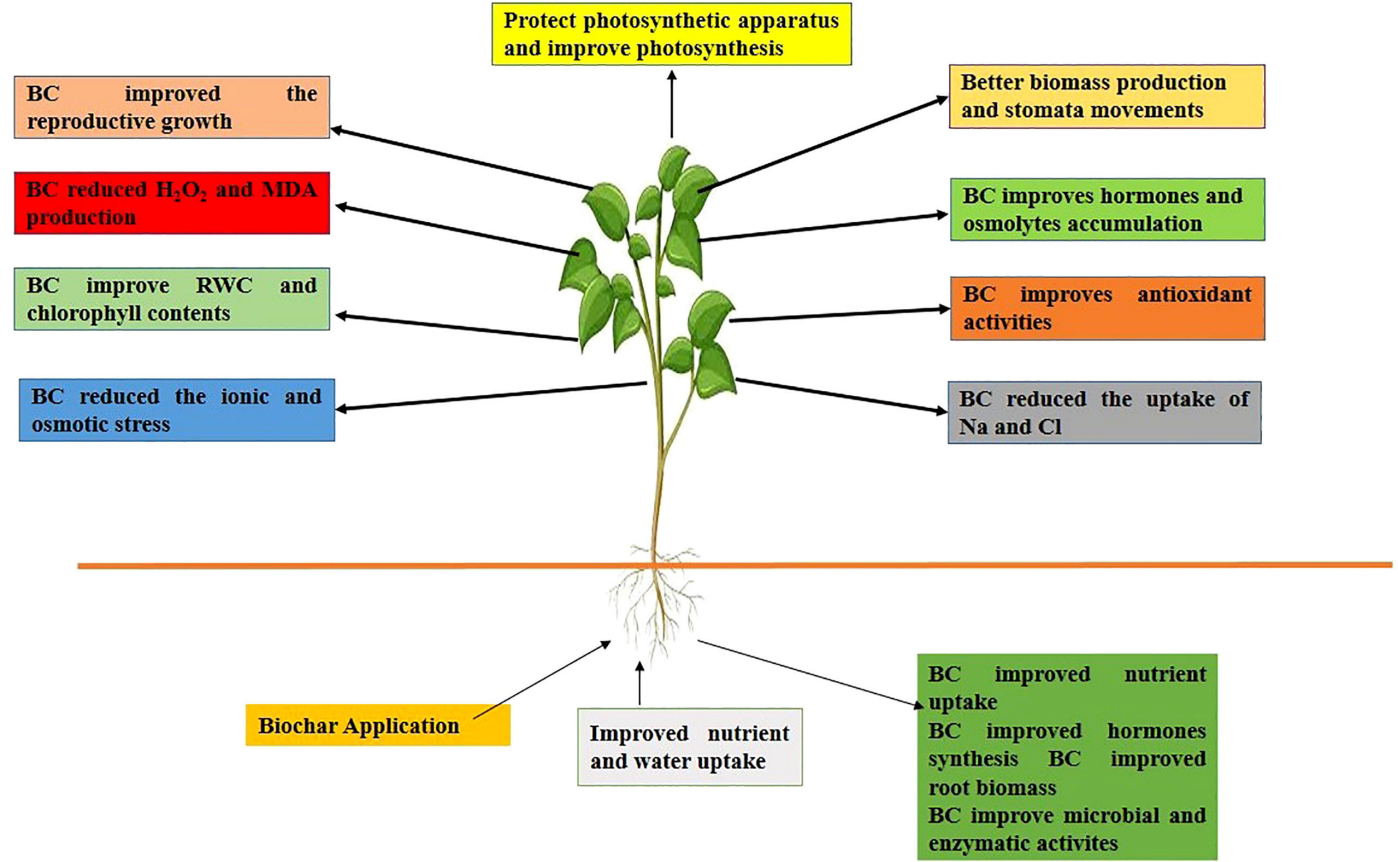
BC application to saline soils improved nutrient and water uptake, soil physiochemical and biological properties, SOM which improved the growth and yield by improving antioxidants activity, hormones and osmolyte accumulation, chlorophyll synthesis and reducing ionic and osmotic stresses.

The use of biochar is a well-recognized practice to mitigate the effects of salinity stress on plants (Hoque et al., 2022b). The application of BC to saline soils improves the growth and yield by improving the uptake of essential nutrients (**Figure 2**) (Ca, Mg, Fe, Zn, Mn, and K), soil porosity, aggregate stability, OM, and decreasing the concentration of toxic ions (Hussain et al., 2019; Ran et al., 2020). Further, the effect of BC on soil properties and plants under saline soils is presented below.

### Biochar improves soil physico-chemical properties to counter salinity stress

Biochar being a soil amendment has got significant attention across the globe. The application of BC to mitigate the salinity stress by improving soil physical properties, water holding capacity, bulk density, CEC, microbial biomass carbon, and enzymatic activities (**Table 3**) (Lehmann and Joseph, 2009; Sohi et al., 2010). Further, BC also adsorbs the toxic ions (Na and Cl) owing to its high surface area and CEC which reduce the toxic effects of salinity (Thomas et al., 2013). Furthermore, BC application also improved the growth, yield, and quality of crops by reducing Na uptake and increasing uptake of Ca, Fe, K, Mn, and P (Abd-El-Mageed et al., 2020). In another study, it was found that BC in combination with tea compost ameliorates the toxic effects of salinity on wheat by decreasing soil electrical conducitiity (EC), exchangeable sodium percentage (ESP), and sodium absorption ratio (SAR: Bayoumy et al., 2019; Bello, 2021). BC-mediated increase in nutrient uptake under saline soils is linked with a concomitant increase in CEC, soil porosity, and aggregate stability (Zheng et al., 2018).

**Table 3:** Effect of biochar application on soil physiochemical and biological properties of saline soils.

Biochar application	Field/lab study	Soil salinity	Effects	References
Grass BC 2 t/ha	Field	EC (μS/cm) (2400)	BC improved soil organic carbon, organic matter, soil bulk density, soil pH, soil porosity and soil NPK.	Zonayet et al., 2023
Wood chip biochar 75 t ha^-1^	Lab study	EC 23.1 dS m^-1^	BC reduced soil pH, soil EC and SAR.	Chaganti et al., 2015
grape pruning residues	Lab study	9 (dS.m^-1^)	BC increased the soil pH, organic carbon, concentration of total nitrogen, phosphorous, solution potassium, sodium, iron, zinc, copper, basal respiration, and nitrifying bacteria frequency.	Moradi et al., 2019
Palmetto biochar	Field study		BC application boosted the soil porosity, soil evaporation, saturated soil water contents, field capacity and decreased soil bulk density.	Liang et al., 2021
alterniflora shoot biochar	Pot study	Soil salt content 0.6 %	BC addition to soil enhanced the SOC, Total Nitrogen, phosphorus, potassium and also increased the MBC, ${\text{NH}}_{4}{+}^{\;}$ , ${\text{NO}}_{3}{-}^{\;}$ , soil sucrose, urease and alkaline phosphatase activity.	Cui et al., 2021
*Eucalyptus polybractea* wood biochar	Lab study	187 (μS cm^-1^)	BC reduced the ${\text{NH}}_{4}{+}^{\;}-\text{N}$ , ${\text{NO}}_{3}{-}^{\;}$ -N, DOC and TDN concentrations.	
Sugarcane bagasse biochar 30 t/ha	Lab study	10 (dS.m^-1^)	BC increased the SOC, SOM, CEC, DOC, and enzymatic activities	Azadi et al., 2021
Wood biochar 45t/ha	pPot study	249 µS/cm	BC mitigated soil EC, soluble Na^+^ and Cl^-^ concentration and increased CEC, SOM, humic acid, TN, TP, regulate the bacterial abundance and community structure.	Huang et al., 2022
Peanut shell biochar 10%w/w	Lab study	6 (dS.m^-1^)	BC improved the SOC, TOC, MBC, urease and fluorescein diacetate hydrolyzing enzyme activity.	Bhaduri et al., 2016
Salix fragilis biochar 4 g/kg	Lab study	1.63 (dS.m^-1^)	BC reduced Na^+^ concentration, bulk density, ${\text{NO}}_{3}{-}^{\;}$ -N, SAR, and increased ${\text{NH}}_{4}{+}^{\;}-\text{N}$ and saturated hydraulic conductivity of soil.	Xia et al., 2020

DOC, dissolved organic carbon; TDN, total dissolved nitrogen; SOC, soil organic carbon; SOM, soil organic matter.

Biochar also improves the NUE in crops owing to its pours structure, aeration, and large surface area which is conducive to the adsorption of NH^4+^, and a reduction in the inhibition of microbial de-nitrification (Liu et al., 2019). Further, BC application also influences the volatilization of N losses from the salt-affected soils. Research reported that BC with high pH (9.6–10.8) increased the NH_3_ volatilization from salt-affected soils while in sandy soils BC with pH (3.9) reduced the NH_3_ volatilization (Esfandbod et al., 2017). Therefore, BC with low pH can reduce the losses of NH_3_ from saline soils. The addition of BC to saline reduces the bulk density and increases the permeability, soil porosity, soil structure, and hydraulic properties (Chaganti et al., 2015; Jin et al., 2019) and decreases the SAR which mitigates deleterious impacts of salinity (Choudhary et al., 2011). Meanwhile, many authors also found a substantial increase in nutrient uptake (NPK), soil carbon contents, and microbial activities which favored plant growth (Haider et al., 2017; Naeem et al., 2017). However, these effects largely depend on BC application rate, type of feedstock and soil properties and it has been noted high rate of BC could induce N immobilization owing to an increased C/N ratio (Nguyen et al., 2018). In the study, it was found that total mineral N content increased with BC rate from 10 to 30 t ha^-1^ probably due to liming of BC on N availability.

BC improves saline soil structure through its impact on soil aggregation and through improved above and below-ground biomass which consequently affects microbial activities and root zone processes (Fletcher et al., 2014; Kolton et al., 2016). Ca increases aggregate stability and facilitates the N leaching through soil profile and an increase in Ca content through BC application can help to reduce the Na availability and improve the soil’s physical properties (Clark et al., 2007). For instance, Chaganti et al. (2015) conducted a series of lab and column leaching studies and found that BC application aggregate stability and hydraulic conductivity of saline soils by increasing the Ca concentration. Likewise, other authors also found a significant increase in Ca concentration in saline soils following BC application corresponding increase in aggregate stability, hydraulic conductivity, and water retention Amini et al., 2016; Kim et al., 2016). Given that the concentration of Ca depends on feedstock and pyrolysis in temperature and all types of BC are not effectively improved soil properties. Biochar application also improves the soil organic carbon in salt-affected soils (Bhaduri et al., 2016). In a study, Kim et al. (2016) found a substantial increase in the percentage of water stable aggregated following BC application owing to an increase in soil carbon and a decline in ESP. Although farmyard, and poultry manures and compost soil carbon decrease the ESP, nonetheless, organic substances present in BC are slow to degrade which makes him an important amendment for saline soils (Chaganti and Crohn, 2015; Kim et al., 2016). In another study, Chaganti et al. (2015) found a decrease of EC of saline soil by 84, 83, and 82% following the application of BC, bio-solid compost, and green waste as compared to control owing to the leaching of salts. In conclusion BC improves SOM, soil carbon and reduce the ESP and Na uptake, depending on application rate, type of feedstock and soil properties.

### Biochar improves soil biological properties to counter salinity stress

Soil salinity negatively affects microbial growth and enzymatic activities (Egamberdieva et al., 2010). Biochar is an important soil amendment that can significantly improve the soil microbial activity and soil organic carbon in saline soils (Abo-Elyousr et al., 2022). The application of BC to saline soils improves dehydrogenase activity, enhanced soil microbial biomass carbon (MBC), and OM which improves nutrient absorption in saline soils (Abo-Elyousr et al., 2022). MBC is an important indicator of changes in soil organic carbon concentration and decomposition. Thus, any material that alters the soil’s organic carbon affects the activity, microbial community, and diversity. Biochar application to salt affects soils and improves the soil microbial activity by increasing aggregate stability, water retention, and nutrients release for microbes, stimulating the root exudation of dissolved organic carbon and N that are involved in microbial metabolism, decreasing salinity stress, and increasing the provision of carbon for soil microbes (Brewer and Brown, 2012; Jaafar et al., 2014; Gul et al., 2015; Bhaduri et al., 2016; Zheng et al., 2017). However, some authors also found a non-significant impact of BC on MBC (Castaldi et al., 2011; Zavalloni et al., 2011) and even a decrease in soil MBC following BC application in saline soils (Dempster et al., 2012; Chaganti et al., 2015). These controversial results could be ascribed due to the type and properties of feedstock, and the pyrolytic conditions of BC production. For instance, BC produced at high temperatures may contain recalcitrant C which is unlikely to be an energy source for microbes (Lehmann et al., 2011; Song et al., 2014). Hence, feedstock quality, and production procedures could lead to different BC properties that affect the soil ecology and biochemistry however, further studies are direly needed to evaluate the effect of BC on soil health and soil microbes.

### Biochar improves soil enzymatic activities to counter salinity stress

Biochar is an important organic amendment that improved the activity of different enzymes docosahexaenoic acid (DHA), alkaline phosphatase (ALP), and catalase (CAT) therefore reducing deleterious impacts of salinity and improve the yield and chemical and biological properties of soils (Taheri et al., 2022). In another study, it was found that BC application to saline soils increased the MBC, and activity of invertase, urease, and phosphatase (Bahadori-Ghasroaldashti and Sepaskhah, 2022). Similarly, Song et al. (2022) found that BC increased the proline (Pro), CAT, and sucrose (Sur) activity by 13.9%, 8.4%, 21.7%, 81.3%, and 150.5%, as compared to control conditions (Song et al., 2022). The various types and concentrations BC were found to improve the activity of urea, invertase and dehydrogenase under saline soil (Jia et al., 2017; Abou-Jaoude et al., 2020). Similarly, Yao et al. (2021) found that BC supplementation to saline soil increased the CAT, alkaline phosphatase activity, and urea and sucrose activity with a corresponding increase in rice biomass and grain yield. Further BC application also reduced the Na^+^/K^+^ concentration and increased the rice growth and yield in saline-sodic soil (Yao et al., 2021). Moreover, Premalatha et al. (2022) found a reduction in growth and enzymatic activities at high salt stress, however, BC addition mitigated these adverse impacts and improved the growth and enzymatic activities (Premalatha et al., 2022). In another study, BC applied at a rate of 3% promoted the nutrient uptake, soil fertility, and activity of urease and alkaline phosphatase which mitigated adverse impacts of salinity and improved the soil quality and plant growth (Cui et al., 2021). These are the limited studies conducted in the literature to determine the impacts of BC on soil enzymatic activities under saline soils. Therefore, more studies are direly needed to determine the impact of BC on soil enzymatic activities considering the feedstock type and pyrolysis conditions.

### Biochar mitigates toxic ions uptake to counter salinity stress

In saline soils, the concentration of Na^+^ is significantly increased which impaired the uptake of K and other essential nutrients. However, BC averts this condition and improves the uptake of K under saline soils. For instance, Lin et al. (2015) noted that BC application (16 Mg ha^-1^) in saline soil increased the exchangeable K by 44%. The pH of salt-affected soils is > which decreases P availability, however, BC application can increase the availability of P in salt-affected soils because of its inherent capacity to increase P. Also, BC increases the availability of P by increasing the growth of soil bacteria (*Flavobacterium*, *Pseudomonas* and *Thiobacillus*) which solubilize the unavailable P present in soil (Yao et al., 2017; Ding et al., 2020). The application of BC to saline soil reduced Na uptake owing its appreciable adsorption capacity and decreasing osmotic stress, soil moisture, and nutrient concentration (Akhtar et al., 2015a).

Furthermore, in salt-affected soils, BC traps excessive Na in soil and releases the essential nutrients, and decreases the osmotic stress (Ibrahim et al., 2021). BC also reduced the N concentration and Na/K ratio however, it depends on feedstock type and pyrolysis conditions (Lin et al., 2015; Ali et al., 2017). In a research study, BC application directly reduced the SAR by increasing Ca^2+^ and Mg^2+^ in soil. Further BC application also decreased the Na concentration in soil by increasing CEC and BC-induced increase in Ca^2+^ in soil solution promotes the displacement of Na from exchangeable sites which reduced the Na concentration in saline soils (Dahlawi et al., 2018).

Biochar has many beneficial impacts and it reduced the SAR and ESP which can improve plant growth under saline soils (Luo et al., 2017; Sun et al., 2017). BC reduced the ESP through different mechanisms, likewise, BC reduced ESP by increasing the Ca that replaces Na in soil solution and BC also increases the surface charge density which increases Ca concentration and reduces the Na availability (Chaganti et al., 2015; Zheng et al., 2017). Moreover, BC also improves the soil porosity that facilitates the Na leaching, therefore, reduce ESP and SAR while BC also increases the partial pressure of CO_2_ in the rhizosphere that mobilizes the Ca from the calcareous soils which reduce replace the Na from the soil colloids (Jalali and Ranjbar, 2009; Di-Lonardo et al., 2017). As SAR value depends on the relative proportions of Na and Ca in soil solution and the content of Na and Ca vary in BC owing to feedstock and pyrolysis conditions which affect the SAR in saline soils (Kim et al., 2016). The high rates of BC application containing elevated Na can increase ESP and SAR therefore, BC must be pre-test for Na concentration before applying to agricultural soils (Sun et al., 2017; Zheng et al., 2017). To summarize, BC substantially improved the uptake nutrients and reduce the Na and Cl uptake which mitigated the deleterious impacts of salinity stress.

### Biochar mitigates support the plant growth under saline soils

BC is rich in carbon material and many studies have found that BC application in saline soils improves, plant physiological and biochemical functions, enzymes (**Table 4**), and hormones activity that decrease the harmful effects of salinity on plants (Farhangi-Abriz and Torabian, 2018b; Huang et al., 2019; Yang et al., 2020). BC incorporation also improves seedling emergence, root and shoot growth, leaf area, and dry matter production under salty conditions (Ibrahim et al., 2020; Ibrahim et al., 2021). Many authors noted that BC application under saline soils significantly improved photosynthetic rate, stomata conductance, and transpiration in wheat, sorghum, eggplant, and quinoa (Huang et al., 2019; Ibrahim et al., 2020; Parkash and Singh, 2020; Yang et al., 2020). Moreover, BC also application also improves the osmotic balance by increasing CO2 assimilation, water holding capacity, stomata conductance, and photosynthetic rate that favors plant growth under saline soils (Yang et al., 2020; Ibrahim et al., 2021).

**Table 4:** Effect of biochar application on growth, yield, physiological and biochemical responses of different crops under saline soils.

Biochar application	Crop/Field/lab study	Soil salinity	Effects	References
Wood BC 5%	Tomato (pot)	0.2 mol/L	BC improved plant water relations, photosynthetic rate, stomata conductance, root length, biomass, water use efficiency, antioxidant activities and reduced ABA synthesis.	Zhang et al., 2023
Peanut shell biochar PSBC	*Suaeda salsa* (pot study)	239 (μS/cm)	BC improved the total biomass, shoot biomass and root biomass.	Sun et al., 2016
Maple residues biochar	common bean (Phaseolus vulgaris L.)	1.35 dSm^-1^	BC enhanced shoot, root dry weight, leaf area, shoot and root length, relative water contents and chlorophyll contents.	Farhangi-Abriz and Torabian, 2018a
Maize straw biochar	Eggplant (pot study)	300 mM	BC application increased plant height, biomass, no. of fruits, Abscisic acid concentration, leaf water potential.	Hannachi et al., 2023
Rice straw biochar	Rice (pot study)	191.3 μS cm^-1^	BC application boosted the anatomical structure of rice seedlings, root length, seedling emergence rate root and shoot biomass and plant height.	Zhang MY et al., 2019
*Spartina alterniflora* shoot biochar	*Sesbania cannabina* (pot study)	Soil salt content 0.6 %	BC amendment enhanced germination, root biomass, shoot biomass, leaf biomass, stem diameter, plant height and nutrients concentration in root, shoot and leaves.	Cui et al., 2021
Modified Peanut shells biochar 4.5 Mg ha^-1^	Rice (Field study)	-	BC application increased root, shoot biomass, rice yield and P absorption rate.	Wu et al., 2019
Mix biochar 175t/ha (cotton straw +peanut shells, + sawdust)	Maize (field study)	1955 µS cm^-1^	BC addition at high rate enhanced dry matter, and plant N, P, and K concentrations.	Yue et al., 2023
Rice husk biochar 30% (w/w)	Rice (pot study)	5.09 dS/m	BC enhanced survival % of seedlings, shoot height, shoot dry matter, active tillers, no. of panicles, length of panicles and grain weight.	Sudratt et al., 2023
Wood biochar 45t/ha	Rice (pot study)	249 µS/cm	BC increased the above ground biomass, spike dry weight and yield.	Huang et al., 2022

dummy

It has been reported that BC application improved chlorophyll synthesis, and maintain leaf water contents while reducing proline, H_2_O_2_, and MDA accumulation (Ekinci et al., 2022; Huang et al., 2022). Moreover, BC also reduced the toxic effects of salinity by lowering the levels of abscisic acid (ABA), and jasmonic acid (JA) hormones and increasing the levels of indole acetic acid (IAA: Farhangi-Abriz and Torabian, 2018c). Further, under saline conditions, BC application also improved the nitrogen content, and nodulation activity of ribulose bisphosphate carboxylase (RuBisCO) and glutamine synthetase (GS), nitrate reductase (NR) and glutamine oxoglutarate aminotransferase (GOGAT) which improve salt tolerance (Farhangi-Abriz and Torabian, 2018a). BC addition also increased the concentration of unsaturated fatty acids which improves membrane (Ndiate et al., 2021), further BC also improved activities of ascorbate peroxidase (APX), CAT, POD, SOD, and glutathione reductase (GR) which protect the plants from salinity-induced oxidative damage (Kim et al., 2016; Akhtar et al., 2015b; Shabbir et al., 2021).

It has been also reported that BC improves stomata conductance and maintains better leaf gas exchange characteristics that improve photosynthesis and subsequent plant growth under saline soils (Akhtar et al., 2015a). BC application also significantly improves antioxidant activities (CAT, POD and SOD) and improved the functioning of ascorbate glutathione (AsA-GSH) cycle that prevents oxidative damage by maintaining the redox balance (Alam et al., 2020; Abbas et al., 2021). Moreover, BC application also improves gene expression and increases the concentration of Ca^2+^ that induce salt tolerance by modifying signaling pathways (Qin et al., 2021). The application of BC also improves the expression of genes (*NHX1*, *HKT1*, and *SOS1*) which leads to a significant increase in salt tolerance (Li et al., 2022; Soliman et al., 2022). Furthermore, BC also improves osmolytes accumulation and maintains hormonal balance which is an important mechanism of BC-mediated increase in salt tolerance (Ghassemi-Golezani et al., 2020). Additionally, BC-mediated improvement in plant growth and development under saline conditions is linked with improved nutrient uptake, microbial activities, CEC, and reduced Na uptake (Mansoor et al., 2021; Soliman et al., 2022). In conclusion improves plant growth in saline soils by improving soil physiological and biochemical properties, plant functioning, antioxidant activities and reducing the uptake of toxic ions.

## Conclusion and future outlook

Soil acidity hinders the uptake of essential nutrients with a corresponding increase in toxic metals which negatively affect soil microbial and enzymatic activities and soil physio-chemical and biological properties. Biochar with appreciable liming material can be used to ameliorate the acidic soils, however, the effects of BC could be varied according to feedstock composition and pyrolysis conditions. The use of BC in acidic soils increased soil pH, nutrient uptake, SOM, and microbial and enzymatic activities which ameliorate soil acidity and supports plant growth.

Though, limited studies are conducted to fully explore the potential of BC to alleviate soil acidification, therefore, more studies are needed to understand the liming and consequent impacts of BC. In literature most of the studies are conducted under lab conditions, therefore, more pilot plot studies are direly needed in acidic soils to further explore the role of BC. Similarly, there is also a paucity of information regarding the comparison of short and long-term BC application to acidic soils as liming materials. The liming effects of BC could also be varied according to soil and climatic conditions, thus more studies are direly needed on a wide range of soil and climatic for the promising future of BC as an important amendment to manage acidic soils.

Soil salinity is also a serious challenge across the globe to crop productivity and global food security. Saline conditions increase the uptake of toxic ions (Na and Cl), reduces the uptake of essential nutrients (Ca, Fe, Mg, N, P, K, and Zn) and it also negatively affect soil pH, soil microbial and enzymatic activities which induce negative impacts on plant growth. In recent times BC has emerged as an excellent organic amendment to alleviate the deleterious impacts of salinity stress. The application of BC improves soil pH, soil microbial and enzymatic activities, nutrient uptake, and SOM and minimizes the accumulation of toxic ions (Na and Cl), soil ESP, SAR and EC which favors plant growth under saline soils. In most of the studies are conducted at the lab scale, and long-term field studies are needed to explore the potential of BC to mitigate saline conditions. Likewise, there is no recommendation about the rate of BC application for saline soils, therefore, field studies must be conducted to determine the rate of BC application in saline soils considering the BC and soil properties. The role of BC in combination with another amendment like gypsum is not studied therefore, it is mandatory to explore the combined effect of BC and gypsum on saline soils.

## Author contributions

Conceptualization: KH, JS and RHL. Writing original draft: KH, JS and RHL. Reviewing and editing: RPL, FR, SS, CW, GH, SA, MH and MA. All authors contributed to the article and approved the submitted version.
